# Immunological Considerations for the Development of an Effective Herpes Vaccine

**DOI:** 10.3390/microorganisms12091846

**Published:** 2024-09-06

**Authors:** Mahmoud Singer, Mohamed I. Husseiny

**Affiliations:** 1 School of Medicine, University of California Irvine, Irvine, CA 92617, USA; 2 Department of Translational Research & Cellular Therapeutics, Arthur Riggs Diabetes & Metabolism Research Institute, Beckman Research Institute, City of Hope National Medical Center, Duarte, CA 91010, USA

**Keywords:** vaccine design, herpes simplex virus (HSV), virus latency, innate immunity, adaptive immunity, CD8 T cells, memory T cells

## Abstract

Research is underway to develop a vaccine to prevent and cure infection from herpes simplex virus (HSV). It emphasizes the critical need for immunization to address public health issues and the shortcomings of existing treatment options. Furthermore, studies on the HSV vaccine advance the field of immunology and vaccine creation, which may help in the battle against other viral illnesses. The current lack of such a vaccine is, in part, due to herpes viral latency in sensory ganglions. Current vaccines rely on tissue-resident memory CD8^+^ T cells, which are known to provide protection against subsequent HSV reinfection and reactivation without correlating with other immune subsets. For that reason, there is no effective vaccine that can provide protection against latent or recurrent herpes infection. This review focuses on conventional methods for evaluating the efficacy of a herpes vaccine using differential CD8^+^ T cells and important unaccounted immune aspects for designing an effective vaccine against herpes.

## 1. Introduction

To date, there are no approved vaccines against herpes viral infection. Human herpes simplex virus type 1 (HSV-1) and type 2 (HSV-2) are highly infectious and cause human disease. Globally, 3.7 billion individuals are infected with HSV-1 infection, while HSV-2 infects about 500 million individuals [1,2]. Most HSV-1 infections are oral; however, between 122 million and 192 million people are estimated to have genital HSV-1 infection [1]. Genital herpes infections are caused by either HSV-1 or HSV-2, whereas ocular herpes is caused mainly by HSV-1 [3,4]. Close contact with an individual shedding the herpes simplex virus (HSV), typically through saliva, genital secretions, or a mucosal surface, is how the virus is transmitted. The virus can infect vulnerable surfaces such as the throat, cervix, eyes, or minor skin abrasions. Kissing and engaging in sexual activities are typical methods of HSV transmission [5]. HSV-1 is mainly spread through oral contact, while HSV-2 is primarily transmitted through sexual contact. Transmission of HSV-1 to the genital area is possible through oral–genital contact, resulting in the manifestation of genital herpes [6,7]. After first infecting mucosal sites, the herpesvirus moves through the peripheral nervous system, settles in the neurons of the sensory ganglia and initiates latent infection [8]. To minimize the chances of spreading the virus, individuals with herpes can refrain from close physical contact when experiencing an outbreak [9]. Herpes infection is associated with life-threatening encephalitis [10], Alzheimer’s disease [11], blindness [12], and cervical cancer.

The goal of this study is to better understand the immunologic processes underlying responses to herpes vaccines. This can be achieved by examining the phenotypic and functional characteristics of memory and tissue-resident T-cell subsets and the ignored immunological aspects required for a successful vaccine against herpes.

## 2. Overview of HSV Route of Infection and Clinical Complications

The family of herpesviruses is composed of more than 100 viruses of which 8 infect only humans. These are herpes simplex virus types 1 and 2, varicella-zoster virus, human herpesvirus 6, human herpesvirus 7, human herpesvirus 8, Kaposi’s sarcoma virus, cytomegalovirus, and Epstein–Barr virus. Within specific tissues, all herpesviruses have the ability to establish latent infection [13].

### 2.1. Transmission of the HSV Infection

**Figure 1:**
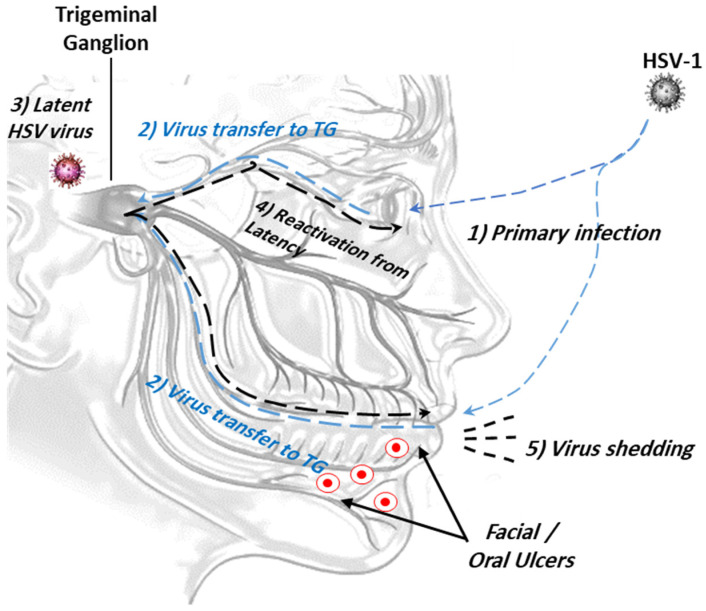
The ocular route of HSV-1 infection from the mouth and/or eye to viral latency in trigeminal ganglion. (1) Primary viral infection and replication; (2) retrograde viral transmission to trigeminal ganglion (TG); (3) viral latency in sensory neurons/trigeminal ganglion; (4) reactivation of latent viruses; (5) reinfection to oral mucosa and virus shedding.

This occurs through intimate contact between the mucosal surfaces of the virus carrier and a non-infected person. Upon initial contact, the virus starts to replicate and is subsequently transported retrogradely by neurons to the dorsal root ganglia, where viral replication and latency begin (Figure 1). Aggressive viral replication may induce severe ulcer lesions and may be life-threatening. The factors that promote the reactivation of latent viruses are still unknown [14,15].

### 2.2. Ocular Herpes Infection

Ocular HSV-1 infection leads to complications ranging from blepharitis, retinitis, corneal ulcers, and blindness [12]. HSV-1 virus usually spreads via airborne droplets and direct contact. Local and systemic manifestations of herpes infection also include encephalitis, retinal necrosis, iridocyclitis, conjunctivitis, and genital herpes, all of which impact quality of life [16,17]. In addition, HSV-1 was found to infect genital organs and cause genital HSV infection contributing to pharyngitis, gingivostomatitis, keratitis, and labialis [17,18,19].

### 2.3. Genital Herpes Infection

HSV-2 is a sexually transmitted virus and can cause a range of symptoms from genital ulcers to severe neurodevelopmental disability and mortality [20]. A subset of HSV-2-positive individuals develop genital herpes with painful genital vesicular lesions and ulcers [17]. The condition is incurable and recurrent in some individuals. HSV-1 is traditionally associated with labial herpes (cold sores), but it can also cause genital herpes through oral–genital contact. Antiviral drugs may be indicated to treat HSV infections, but they cannot eradicate the virus within ganglion cells [21].

### 2.4. Neonatal Herpes Infection

The transmission of the herpes infection from the mother to the fetus/newborn can result in three types of neonatal infection: intrauterine infection (5% of cases), postnatal infection (10% of cases), and perinatal infections (85% of cases). Neonatal instances can appear in a variety of ways. Some infants may only have skin, eye, or mouth disease, while others may have broad infection dissemination or central nervous system (CNS) involvement. [22]. Approximately 30% of neonatal herpes cases progress to CNS disease, while around 25% develop a disseminated disease. The remaining 45% primarily experience skin, eye, and mucosal disease. Long-term disability is more common among those who have CNS and disseminated disease [23].

Mucosal membranes line various cavities and surfaces throughout the body and are classified by their physical barrier properties into permissive, effector permissive, and restrictive [24]. Permissive tissues support viral replication and are readily accessible by immune cells, even in the absence of local inflammation or the presence of an antigen, including the spleen, lung, liver, kidney, and adipose tissue [25,26]. Effector permissive membranes are accessible by effector immune cells but not memory immune cells. Through the presence of an antigen or inflammation, effector and memory T cells migrate to these tissues and colonize them. Once the infection is resolved, these tissues become inaccessible to circulating memory T cells, and examples are the gut, brain, and peritoneal cavity [27,28]. Restrictive tissues include the skin, vaginal epithelium, salivary glands, lung airways, ganglia, cornea, and sensory ganglia. These tissues are inaccessible to effector or memory T cells in a steady state and are only accessible to effector T cells during local inflammation [27,29]. Beyond protective immune barriers, the main immune cell defense for protecting mucosal tissues against exogenous antigens and pathogens are resident memory (T_RM_) and effector memory (T_EM_) T cells [30].

## 3. Mechanism of Viral Latency

During the acute phase (3 to 10 days post-infection), the virus can be readily identified within the sensory ganglia but rapidly disappears due to the adaptive immune response. Viral reactivation after latency is usually accompanied by clinical complications of the disease [31]. Latency of HSV-1 in the sensory neurons is thought to be due to the failure of the IE gene or VP16 expression and impairment in the initiation of the lytic cycle [32]. In sensory neurons, the reactivation of the latent virus is mainly dependent on efficient lytic cycle gene expression, which, in turn, relies on the transactivating function of the VP16-induced complex that is formed by the structural proteins VP16, HCF-1, and Oct-1 [33]. Studies on wild-type HSVs show many alternative pathways linked to latency that involve viral DNA delivery into the neuronal cell nucleus. Therefore, latency is achievable by mechanisms that do not require a complete block of viral gene expression and rely on viral transcripts [32]. The research suggests that viral latency increases the risk of cancer by inducing spontaneous mutations that result in chromosomal rearrangements, substitutions, insertions, or deletions [34,35,36].

The latency-associated transcript (LAT) of HSV and its associated microRNA play a role in the accumulation of viral lytic gene transcripts and limit HSV-IE gene expression [37]. Moreover, the post-translational modification of histones associated with HSV promoters and the LAT intron are strongly associated with the control of latency [38,39]. The lack of vaccine effect is no doubt secondary to virus latency in neurons where immune cells and antibodies cannot gain access [40].

One of the HSV characteristics that may lead to failure in finding an appropriate vaccine is the presence of the outer shell tegument protein, which bears a more disordered outer shell of the virus. This phenomenon occurs in HIV as well. HSV-2 has a slightly more disordered tegument than HSV-1, making it difficult for effector immune cells and antibodies to discover the virus with acceptable affinity. For that reason, the discovery of a successful HSV-2 vaccine is a harder challenge [41,42].

## 4. Immune Reactions against HSV

### 4.1. Innate Immunity

The innate immune system serves as the initial line of defense in eukaryotic organisms. The anatomical barrier-forming structural component and the chemical component are the two primary parts of the innate immune system [43]. Viral infection initiates an innate immune reaction by viral molecules, like DNA, RNA, and glycoproteins. Innate immune cells first recognize pathogen-associated molecular patterns (PAMPs). Toll-like receptors (TLRs) are expressed by innate mononuclear immune cells, including dendritic cells (DCs), natural killer (NK) cells, and NKT cells, and are increased as part of the innate immune response [44,45]. The immune cells have TLR ligands that inhibit HSV-2 replication in genital herpes, indicating the function that TLRs play in immune defense against herpesvirus [46]. Type I interferon (IFN) is produced by the IFN-α1 transgenic pathway that triggers RNA-dependent protein kinase (PKR) in response to viral identification, ultimately leading to an antiviral state [47]. It has been found that NK cells help lower viral loads and improve DC stimulatory ability. Activated NK cells can restore deficient CD8^+^ T cells generated on their own and make up for the loss of CD4^+^ T cells [48]. Despite being widely documented, the interaction between innate immunity and various viral diseases is not taken into consideration in the design of new vaccines.

### 4.2. Adaptive Immunity against HSV

Current vaccines target adaptive immunity, specifically T cells and their effector cytotoxic potential, and long-term memory cells to eliminate HSV or generate neutralizing antibodies [49]. The innate immune response is the important trigger for the adaptive immune response. Adaptive immunity consists of cellular and humoral immunity. The main target of the adaptive immune response is to remove pathogens and generate long-term memory immune cells.

### 4.3. Cellular Immunity

The crosstalk between CD4^+^ and CD8^+^ T cells is essential for viral clearance and is required by the HSV-2-specific immune response [50]. CD4^+^ T cells contribute to the effectiveness of therapies against HSV-2 [51]. However, CD8^+^ T cells are the only T cells that persist at the dermal–epidermal mucosal junction (DEJ) [52]. DEJ tissue-resident CD8a^+^ T cells are responsible for immune surveillance and the initial repression of HSV-2 reactivation in human peripheral tissue [53]. HSV antigens affect the IFN-γ production of various memory T-cell subsets. CD8^+^ T cells exclusively produce IFN-γ, whereas memory CD4^+^ T cells produce both IFN-γ and TNF-α [54].

Regulatory T cells (Tregs) play a role in HSV-2 infection. High levels of interferon were detected in the draining lymph nodes and decreased at the site of infection in Treg-depleted mice. Moreover, the absence of Tregs affected T cells, NK cells, and DCs trafficking to the infection, which was accompanied by elevated levels of pro-inflammatory chemokines. Thus, Tregs promote immune cell migration into infected tissue and constitute an early protective response against HSV infection [55].

### 4.4. Humoral Immunity

B cells are immune cells that contribute to viral clearance by the secretion of antigen-specific antibodies (IgG and IgA) against HSV [51]. In some situations, vaccines are ineffective despite stimulating the production of HSV-2-specific antibodies [56]. In contrast, the HSV vaccine drives B cell antibody production and protects against HSV-1 [56]. B cells and DCs are synergistically induced via IFN-γ secretion by CD4^+^ T cells [57]. The pre-challenge level of pan-HSV-2 IgG correlates with the decline in HSV-2 viral shedding and an improved survival rate [58].

A vaccine based on the fusion of the gD2 and IgG Fc fragments showed long-term effective mucosal and systemic immune protection against HSV-2 [59,60]. HSV-specific IgG was the primary factor that inhibited viral pathogenesis in cerebrospinal fluid (CSF) [61]. Collectively, B cells and antibodies are important in protecting against HSV infection. Further preclinical research is required to define the mechanism behind B cell and antibody participation in humoral defense.

### 4.5. Cytokines

Cytokines have either positive or negative impacts on immune reactions. For example, IFN-α and IFN- l support resistance against genital herpes infection [51,62]. Conversely, IL-15 is essential for NK- and NKT-cell innate immune responses [63] and mediates TLR responses [64]. The pro-inflammatory cytokine TNF-α is the main cause of death in an animal model of herpes infection, and a TNF-α antibody reduce death in mice lacking CXCL10 [65].

Several cytokines and chemokines are secreted from non-immune cells and show a protective effect on herpes infections. Keratinocytes secret TNF, IL-1, IL-6, IFN-a/b, CCL5, CXCL9, CXCL10, CCL20, and CCL27, which play a protective role against HSV through reducing viral spread in the keratinocytes and facilitate the recruitment of CD8^+^ T cells and Th17 T cells [66,67,68].

## 5. Current Approaches for Design of HSV Vaccines

Out of nine Herpesviruses family members, herpes simplex viruses are the alpha domain members of this family [69]. The herpes simplex virus (HSV) genome contains more than 84 proteins that are encoded by over 90 unique genes [70]. The classification of these proteins is traditionally based on their expression timing, with three classes identified: immediate-early (α), early (β), and late (γ). The virus prioritizes the encoding of immediate-early (α) genes, as their products are vital for the expression of the subsequent group of genes. These genes include ICP0, ICP4, ICP27, ICP22, and ICP47. Early (β) genes encode proteins that are mainly involved in viral DNA replication. The late (γ) genes produce proteins that play a role in the assembly and release of virions. HSV possesses glycoprotein spikes on its envelope, which are partially encoded by both the virus and the host’s nuclear membrane. The glycoproteins included are gB, gC, gD, gH, and gL [71].

Currently, there is no vaccine available for HSV-1 and HSV-2, but there are multiple vaccine candidates being developed that give some hope. These vaccines are being created to target both prevention and therapy, and some may have applications for both purposes [42,72,73]. A trivalent surface antigen vaccine contains HSV-2 glycoproteins C, D, and E [74]. Protein subunit vaccines are safer than live-attenuated vaccines but provide only a short-term immune response [42]. The multivalent DNA vaccine SL-V20 was tested on mice and reduced clinical signs of infection. A nucleoside-modified mRNA vaccine could be the next step in vaccine development [74]. Adenovirus-based vaccines are recombinant vaccines, such as rAd-gD2ΔUL25 and rAd-gD2 + rAd-ΔUL25, and are shown to increase survival rates and reduce viral replication [60]. Other vaccine candidates include HerpV and GEN-003/MM2, which are currently in phase I/II clinical trials [75]. 

The most protective and economical method to overcome herpes infection is to find a vaccine against infection and/or reactivation [76]. Several factors for rendering an effective vaccine have been considered, including viral pathogenesis, immune responses to HSV, formulation of the vaccine, adjuvants, and the route of immunization [77]. Approaches for designing and testing potential vaccines conducted in preclinical and clinical stages are listed in Table 1.

A live-attenuated virus is a more potent vaccine approach that is more immunogenic than subunit vaccines and safer for individuals with immune deficiencies. However, some preclinical trials found that live-attenuated viruses, such as ICP0- and gE- HSV-2t, showed protection in animals but were not effective in preventing viral latency [78,79,80,81].

Live-attenuated bacteria, like *Salmonella typhimurium*, are useful carriers for the expression of foreign antigens, including glycoprotein D (gD) and the immediate-early protein ICP27 of HSV-1 [82]. Such vaccines have demonstrated efficacy in eliciting both CD8^+^ and CD4^+^ T-cell-mediated immune responses in models of infectious diseases and cancer [83,84,85,86], as well as controlling autoimmune diseases [87].

Protein-based subunit vaccines are a combination of glycoprotein and viral proteins that can stimulate an immune response. This type of vaccine is safe and effective for some viruses, such as the human papillomavirus, and may inhibit viral entry and shedding, immune-evasive responses, and cell-to-cell transmission [88,89,90,91,92]. Peptide-based vaccines work by targeting immune responses against specific antigens through single or multiple peptide T-cell or B-cell epitopes. This demonstrates better results when combined with bacterial or viral adjuvants and is protective [93,94]. The development of peptide-based vaccines is hindered by the variation in immune responses to peptides among individuals [91,92].

DNA- and mRNA-based vaccines demonstrate moderate efficacy, which is greater than that of subunit vaccines but less than that of live-attenuated vaccines [76,95,96]. Additionally, these vaccines have a higher effectiveness in stimulating the development of neutralizing antibodies [97]. In comparison, adenovirus vector-based vaccines and DNA exhibit a better stability profile, synthesis characteristics, and purification protocol than mRNA vaccines. DNA vaccines are superior in limiting influenza, measles, flavivirus, HIV, and malaria [76,95,98]. This vaccine design approach has been used to treat SARS-CoV2 and HSV [96,99].

There has been a recent development in herpes vaccination, where the activation of tissue-resident memory (TRM) T cells was utilized [100]. This strategy induces systemic T-cell responses, followed by activated T-cell recruitment via chemokines to infected mucosal tissues [100,101,102]. This model, named the “prime/pull mucosal vaccine” concept, was similarly implemented for other infectious diseases [103,104]. This approach is powerful in controlling HSV-2 spread into the sensory neurons and has the ability to recruit more naïve and central memory cells to enhance T_EM_ and tissue T_RM_ cell numbers [100,105]. However, there is no evidence of this type of vaccine providing long-term protection, and there are no data about the interaction of innate immune cells with this approach.

A recent approach has been adopted for a potentially curative therapy for herpes based on gene editing technology, using CRISPR/Cas9 to modify the viral transcript (such as meganucleases or a latent promotor or IL-15) and a delivery system of adenovirus vectors. The benefit of this technology is that it provides unprecedented efficiency in eliminating 90% or more of latent HSV-1 virus DNA and up to 97% of latent HSV-2 DNA in animal models, in addition to decreased viral shedding from infected ganglions. Although this technology shows a promising trend for vaccines and therapy, dose optimization is still required to avoid hepatotoxicity and histological neuronal injuries [106,107,108,109].

**Table 1:** Current HSV-1 and HSV-2 vaccine approaches: different up-to-date strategies and clinical stages.

Classification	Vaccine Design	Year	Stage	Study Model	Benefits	Disadvantages	References
Replication-defective	Deletion of HSV-1 gH coding sequences (SC16∆gH) Deletion of HSV-2 UL5 gene or UL4 ORF (dl5) or UL29 gene or UL5 and UL29 genes (dl5-29) or ICP10∆PK	1994–2019	Preclinical and clinical	Mice Guinea pigs Human	Establishes a self-limiting infection. Protects against acute infection, local viral replication, primary disease, and recurrence and shortens disease episodes. Long-lasting immune responses over 6 months. Better potency for complete protection. Induces defective viral replication and latent infection by reducing viral titer and shedding. Safe for immunocompromised individuals. Induces memory T cells by eliciting HSV-specific T-helper type 1 and increases IL-12 production by DCs. Promotes increased T-cell responses and anti-HSV neutralizing antibody production. Effective against a wide range of HSV strains.	No improvements in duration of viral shedding, frequency and severity of recurrences, and lesion healing time. Non-efficient during first recurrence of genital HSV. Induces delayed-type hypersensitivity responses. Some of the antigens only induce CD4^+^ T cells in HSV-seropositive individuals.	[110,111]
Replication-defective	HSV-2 ICP8− with B7 co-stimulation molecules Deletion of HSV-2 glycoprotein D	2007–2020	Preclinical	Mice	Boosts FcγR-activating responses and increases IgG2 antibodies. Increases effector T-cell production of IFN–γ. Decreases viral replication and spreading in mucosa and to the sacral ganglia. Improved overall survival.	Patients still show signs of genital and neurological disease. Not applicable for measuring reactivation of latent virus.	[112,113]
Live-attenuated	Live rHSV (R7017, R7020, RAV9395, VC2 with mutations in gK, membrane protein, R2) HSV-1 0∆NLS, HSV-2 0gD∆NLS	1998–2020	Preclinical	Mice Guinea pigs Rabbits	Reduces viral shedding and recurrent disease in ocular and vaginal herpes, TG, and brain neurons. gB induces expression and release of IFN-γ, granzyme B, and CD107a and decreases T-cell exhaustion (LAG-3, PD-1, and TIM-3). Protects against severe infections and lethal IV antigen challenge.	Option as a prophylactic vaccine, not as a therapeutic vaccine. Not sufficient to provide broad protection against HSV infection.	[72,76,78,114,115]
Protein-based subunit	gD2t, gD, gB, gE2 (mixed with adjuvants)	2002–2020	Preclinical and clinical	Mice Guinea pigs Human	Protects against acute and recurrent HSV-2. Induces antigen-specific CD8^+^ T cells and high antibody levels and reduces viral shedding. Induces mobilization of DCs. Sustained durability of response for up to 21 months.		[79,116,117]
Peptide-based	Neutralizing epitope of CD8, CTL, and T helper HLA-A2 epitopes, HSP + 32-35 mer peptides	2011–2021	Preclinical clinical	Mice	Reduces vaginal lesions. Generates high levels of mucosal antibodies (IgA). Able to block viral infection.	Toxicological studies are absent and not tested against latent infections.	[91,92,118]
Naked DNA vaccine	pDNA encoding several genes	1995–2020	Preclinical and clinical	Mice Guinea pigs Human	Decreases viral shedding. Prevents pathological progression after infection, improves survival, and increases infiltration of leukocytes. Induces specific cytotoxic T cells and is safe and well tolerated. Reduces latent viral load.	Limited protection against lethal dose in the animal model.	[76,119,120]
mRNA-based HSV vaccine	Tri-HSV mRNA encoding the ectodomains of gC1, gD1, and gE1 proteins	2023	Preclinical and clinical	Mice Human	Stimulates robust CD4^+^ T-helper cells and germinal center B-cell responses and produces high levels of antibodies. BNT163 (BioNtTech, Mainz, Germany) (ClinicalTrials.gov Identifier, NCT05432583), mRNA-1608 vaccine (Moderna Inc., Cambridge, MA, USA).		[121,122,123]
Prime-pull vaccine	Adv viral peptides + T-cell chemokines (CXCL-x)	2018	Preclinical clinical	Mice Guinea pigs Rabbit Human	Mobilizes tissue-resident and effector T-cell subsets to the site of infection. Can prime with different peptides or adenoviral vectors. Shows humoral and cellular immune activation against active and latent infection.	Toxicological studies are absent.	[118]

CXCL: The chemokine (C-X-C motif) ligand; g: glycoprotein; ICP8: viral single-strand DNA-binding protein; LAG-3: lymphocyte-activation gene; ORF: open-reading frame; PD-1: programmed death-1; pDNA:(plasmid DNA); rHSV: recombinant Herpes simplex virus; TG: trigeminal ganglion; TIM-3: T-cell immunoglobulin domain and mucin domain-3.

## 6. T-Cell Epitope Vaccines

Sub-populations of diverse clones of memory CD8^+^ T cells can be categorized based on differences in their phenotypes [114], effector function, proliferative capacity, anatomical locations, and long-term fate [124,125]. After resolving a viral infection, around 90% of effector CD8^+^ T cells are cleared, leaving 10% behind to transform into memory CD8^+^ T cells [124,125]. The heterogeneity of memory CD8^+^ T cells is important in tracking the developmental lineage of CD8^+^ T-cell subsets [126,127,128,129].

### 6.1. Identification of T-Cell Immunophenotypes to Evaluate T-Cell Vaccines

This analysis is crucial to identify the diverse subsets and functionality of memory CD8^+^ T cells, including CD62L, CD44, IL-7R (CD127), CD69, CD11a, CCR5, CCR7, CD103, and α4β7; IL-2/IFN-γ/TNF- α, perforin, granzymes A/B/C/K, and programmed death-1 (PD-1) for effector functions and/or dysfunction; and Bcl-2, CD122, CD28, CD57, CD27, KLRG1, CXCR3, and CD43 for survival and/or proliferative capacity [130,131]. The expression of these markers mainly relies on (i) the type and duration of infection, (ii) inflammatory cytokines, (iii) Ag-specificity, (iv) naïve T-cell precursor frequency, and (v) location within the body [132,133,134]. CD8^+^ T-cell subsets may exhibit a range of differentiated phenotypes, such as spanning short-lived effector CD8^+^ T cells (SLECs, IL7R^low^ and KLRG1^high^), memory precursor effector CD8^+^ T cells (MPECs, IL7R^high^ and KLRG1^low^) [135,136], central memory T cells (T_CM_) (CD45RA^−^, CD62L^+^, and CCR7^+^), T_EM_ (CD45RA^−^, CD62L^−/dim^, and CCR7^−^), and T_RM_ (CD45RO^+^, CCR7^−^, CD62L^low^, CD69^+^, and CD103^+^) [137,138].

**Figure 2:**
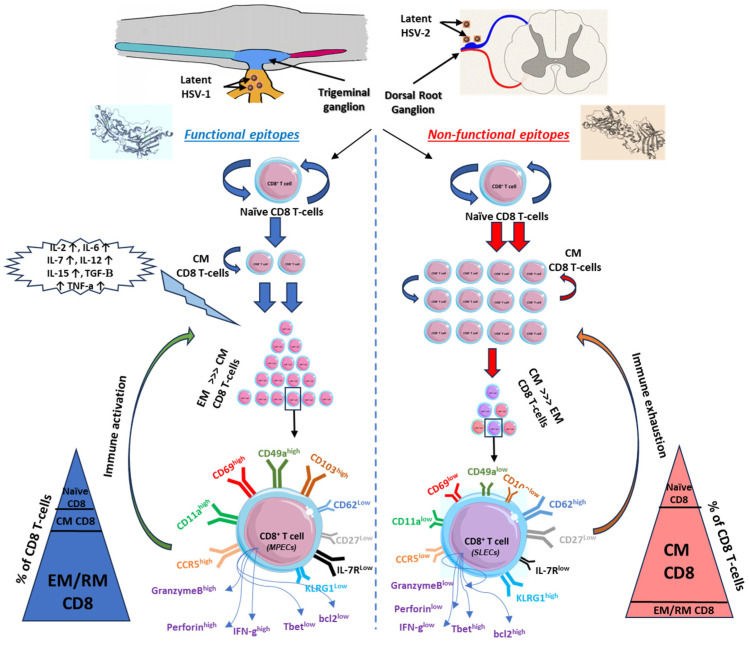
Kinetics of CD8 T cells in relation to functional versus non-functional epitopes. Functional epitopes (**on the left**) appear to induce long-lived effector memory and tissue-resident CD8^+^ T cells that reside within the mucosal tissues and related ganglions compared to non-functional epitopes of herpes antigens, which induce short-lived effector memory T cells (**on the right**). Upon reactivation of functional epitopes, naïve CD8 T cells are matured to central memory by the induction of pro-inflammatory cytokines. EM/RM CD8 T cells show unique phenotypic characteristics that support the function of viral clearance and long-term precursor of effector and memory CD8 T cells (**on the left**). On contrast, non-functional epitopes generated short-lived memory T cells with a major phenotype of central memory more than EM/RM CD8 T cells (**on the right**). Based on the high-affinity recognized antigen, long-lived effector memory T cells are generated from these functional epitopes of the antigen with high frequency compared to central memory or naïve T cells and characterized by higher expression of activity and tissue residency markers (CD103^high^, CD69^high^, CD11a^high^, CD49a^high^, CCR5^high^, and IFN-γ^high^).

The non-functional and functional epitope stimulations are supplemental major factors affecting the expression of phenotype markers of memory CD8^+^ T cells and hence define the fate of antigen-specific memory CD8^+^ T cells. There are two theories advanced to explain intravaginal infection with HSV-2. First, functional epitopes appear to preferentially induce T_EM_ that are destined to survive and become long-lived CD8^+^ T cells that reside within the vaginal mucosal tissues (local site) (Figure 2). In contrast, non-functional epitopes appear to induce central memory non-functional CD8^+^ T cells (systemic) (Figure 2). Second, cytotoxicity hindrance occurs by viral-induced immune exhaustion to effector T cells or immunosuppression induced by Tregs [139,140]. Memory CD8^+^ T-cell heterogeneity in mucosa-cutaneous tissues depends on adhesion molecule expression patterns [141]. For example, in mucosal tissue-resident cells, CD103 (α4β7 integrins), which binds to epithelial cadherin (E-Cadherin), is highly expressed in the memory CD8^+^ T cells of HSV-seropositive asymptomatic patients (Figure 2). This upregulation of CD103 on memory CD8^+^ T cells mediated by TGF-β plays a critical role in the differentiation of memory T cells and rapid infection control in mucosal tissues [142,143].

### 6.2. Functional Assays to Evaluate T-Cell Vaccines

These tests identify the capacity and the subsets of CD8^+^ T cells. Assays, like intracellular secreted cytokines, and some functional markers, like IL7R and KLRG1, assist in subset differentiation. IL-6 is a potent inflammatory cytokine that works with IL-7R to support the functionality of memory CD8^+^ T cells [144]. Polyfunctional CD8+ T cells are potential surviving cells for the creation of MPECs because of their increased capacity to secrete different cytokines. Some SLECs can give rise to a terminally differentiated population of T_EM_ cells [145,146]. Polyfunctional CD8^+^ T cells are responsible for protection against ocular and genital herpes [147].

The inflammatory cytokine repertoire (IL-6, IL-8, and IL-12) during T-cell priming controls the fate of effector CD8+ T-cell development [148], while IL-15 and other accessory factors are necessary for cell survival [128,149]. The expansion and activation of CD8^+^ T cells primarily depend on the balance between inflammatory cytokines, like IL-2, and anti-inflammatory cytokines, like TGF-β [149,150]. It remains unclear as to the mechanisms by which the cytokine/chemokines milieu influences the selection of a heterogeneity model of non-functional and functional epitope-specific memory CD8^+^ T-cell subsets.

### 6.3. Transcriptome Analysis for T-Cell Vaccines

Several mRNA transcripts participate in the maturation of T_EM_ cells [149,151]. Examples include Bcl2, Blimp1, Id2, Id3, Eomes, Tcf1, and T-bet [152]. Particularly, T-bet is a key lineage-determining factor that promotes maturation toward SLECs or MPECs [127,148]. T-bet levels during inflammation change the fate of CD8 T cells, where high T-bet promotes SLECs and low T-bet promotes MPECs [148,153]. The majority of functional CD8^+^ T cells have a phenotype that is tissue-resident. Memory T cells are found in the infection site of mucosal tissues, respond favorably to antigens or homeostatic cytokines (IL-15 and IL-7), and have a reduced ability to migrate to lymphoid tissues [149]. Non-functional CD8^+^ T cells are primarily central memory phenotype cells and lack the appropriate expression for homing to inflammatory sites, such as lymph nodes, migratory molecule L-selectin, and chemokine receptors (CCR7 and CCR5) [154,155].

## 7. Conventional Method for Evaluating Efficacy of Herpes Vaccine by Differential CD8^+^ T-Cell Interactions against HSV

Conventionally, in viral vaccines, T cells are acknowledged for their importance in the immune system by establishing a memory immune response using chemokines and their protection capacity at mucosal surfaces and in viral infections, supporting the importance of T_RM_ cells in the control of mucosal herpesvirus infections [119,156]. Hence, T-cell maturation subsets are used to evaluate the effectiveness of designed vaccines [157].

### 7.1. Resident Functional Memory CD8^+^ T-Cell Epitopes

HSV-2 is a sexually transmitted virus infecting vaginal mucosal (VM) tissues [100]. At a steady state, effector and memory CD8^+^ T cells are inaccessible to restrictive VM tissues, and cells can gain access only under the local inflammation of primary or recurrent herpes infection [100]. A small subset of CD8^+^ T cells reside in the DEJ of the VM tissue where latent HSV is released during reactivation from infected neurons of the sensory ganglia [53]. These resident CD8a^+^ T cells in the DEJ tissue are responsible for the early containment of HSV-2 reactivation in the infected tissue [53]. The generation of protective VM-resident memory CD8^+^ T-cell immunity against sexually transmitted HSV depends on the development of long-lasting functional CD8^+^ T_EM_ cells [127,149]. One of the unique benefits of mucosal immunization is that it induces systemic and mucosal immune protection [158]. Studies show the prime/boost VM vaccine (Lipo/rAdv5) induced robust long-lived HSV-specific functional CD8^+^ T cells that protected against recurrent infection [159]. Compared to other mucosal surfaces, VM tissues have more complicated intrinsic characteristics that support the growth, survival, and retention of functional CD8+ T_EM_ cells [100].

### 7.2. Types of Memory CD8^+^ T Cells

Memory CD8^+^ T cells are categorized into three major subtypes: T_CM_, T_EM_, and T_RM_ cells [160,161]. T_CM_ cells are mainly located in the periphery and lymphoid tissues [143,162]. They are high in proliferation, secret IL-2 upon re-stimulation [163,164,165], and display CD62L^high^, CD44^high^, IL-7R^high^, and CD103^low^ [143,154,166]. Upon activation with viral antigens, they undergo terminal differentiation for cytotoxic effector functions [143]. T_RM_ cells are mainly located in retained tissues within the portal entry sites of potential invading pathogens that provide rapid long-term protection against tissue re-infection [143,162]. T_EM_ cells are recognized by the downregulation of T-cell homing molecules (CD103^high^, CD62L^low^, and CCR7^low^) and upregulation of nonlymphoid homing adhesion molecules and chemokine receptors [167,168]. A skin-resident HSV-specific CD8^+^ T_EM_/T_RM_ cell subset persisted up to 8 months after viral infection [141]. In contrast to T_CM_, T_RM_ cells express CD62L^low^, CCR7^low^, CD11a^high^, CD69^high^, CD103^high^, and CD49a^high^ [160,165]. Furthermore, T_RM_ cells constitutively express high levels of granzyme B and eliminate infected target cells with secreted perforin [138,163]. The similarities between both T_EM_ and T_RM_ cells are that they differentiate and reside in extra-lymphoid tissues and have an immediate effector function [169]. T_RM_ cells are remarkable as they produce pro-inflammatory antiviral cytokines, like IFN-γ, TNF- α, IL-22, and IL-17, and chemokines, such as MIP-1 [170].

## 8. Important Perspectives of Immune Aspects for Designing a Successful Vaccine against Herpes

Scientists are suffering from ineffective herpes vaccinations. Current herpes simplex vaccines focus on T cells and B cells for developing cellular and humoral immune responses, respectively [171]. Additional immune cell subsets should be considered when designing an effective herpes vaccine. An intersection of innate immune pathways with the latent HSV genome was documented [172]. The absence of overlap between innate and adaptive responses is one of the weaknesses of the herpes vaccine research. Monocytes, macrophages, and B cells are considered as viral reservoirs for viruses. Rather than serving as a vehicle for viral antigens to reach effector cells, they are tricked and turn into a target for the virus to hide and spread [173,174].

Mononuclear phagocyte cells, including CD11b^+^, Ly-6C^+^, and Ly-6G^low^ monocytes, CD68^+^ macrophages, and CD11c^+^, CD1c+, and MHC-II^+^ DCs, participate in cellular defense against HSV. Immature and mature DCs are permissive in viral replication, while lytic HSV infection is encouraged by immature DCs [175,176]. DCs and macrophages have the capacity to trap and retain viruses. This capacity raises the question of whether DCs can be infected by cell-to-cell contact with other adjacent cell types [175]. HSV-1 and HSV-2 induce DC paralysis by interfering with adhesion molecule expression, such as LFA-1 and CD83 [177]. Monocytes carrying engineered HSV were tested in cancer models [176]. Mice implanted with melanoma and infected with HSV showed Treg depletion, while patients treated with depleting CD25 cells showed changes in T-cell dynamics [178]. However, in transgenic mice with ocular HSV-1 infection, the infiltration of CD4 T cells resulted in homeostatic expansion and worsening of the disease [179]. CD4 T-cell migration to the site of infection and the subsequent phenotypic changes alter cell functionality via MALAT1-mediated immunosuppression [180] or activation by granzyme B [181].

Monocytes and macrophages are the main sources of IL-1β during infection or stress expressed and released throughout the body upon inflammation [182]. IL-1 receptor-type 1 (IL-1R1) activation typically leads to an inflammatory response or antiviral reactions in most cell types. Monocytes and macrophages are known to be viral targets and vessels for dissemination. Long-term viral latency and viral genome persistence within tissues are intricately connected to the lineage of monocytes and macrophages. In response to herpes infection, monocytes display heightened levels of proinflammatory signaling molecules and initiate antiviral responses [174]. Latency is achieved through the combined effect of immune suppression mechanisms and herpesvirus infections. In order to evade the host’s innate immune system, HSV-1 has evolved multiple mechanisms that suppress host antiviral elements, enabling efficient infection [183].

NK cells and plasmacytoid DCs (pDCs) are involved in the innate immune response against HSV. Toll-like receptor 9 on pDCs allows them to recognize the herpesvirus DNA found in endosomes. In response, they release large amounts of type I interferon, which prevents the infection from spreading throughout the body. Interferon binds to NK cell receptors, activating cells and allowing them to eradicate virus-infected cells [184,185,186].

Bortezomib, a proteasome inhibitor, impacts the lytic cycle of herpesviruses and influences latent HSV-1 genomes to increase reactivation. This occurs independently of any effects on the immune response. Nevertheless, a reduction in CD11b^+^, Ly-6C^+^, and Ly-6G^high^ systemic neutrophils might increase the risk of adverse outcomes. This emphasizes the relevance of neutrophils in controlling HSV-1 infection. IL-36γ released by the epithelial mucosa recruits neutrophils to herpes-infected reproductive tissues and protects neurons [187].

In mucosal tissues, mast cells (MCs) are involved in allergic reactions as well as pathogen protection and monitoring [188]. MCs promote inflammation in ocular allergies. They regulate the influx of polymorph mononuclear leukocytes, which inhibits viral replication and reduces inflammation. They act as a reservoir for supplemental viral replication [189]. MCs can increase leukocyte adhesion molecule expression and vessel permeability [190,191]. During viral infection, they produce type I interferon and degrade inflammatory mediators by MC protease 4 [192,193].

Brain microglia constantly survey their microenvironment for pathogens and, using pattern recognition receptors, coordinate the innate immune response. Microglia in the choroid plexus employ STING and interferon against HSV-1 [194]. IFNs are produced in response to retinal necrosis, triggering the activation of the innate immune response. With a loss of IFNs, the virus can spread to nearby and distant tissues faster [195]. Type 1 IFN boosts the function of NK cells and regulates IFN-γ, CD4^+^, and CD8^+^ T cells. Nonetheless, the type 1 response can be affected by pre-existing or concurrent type 2/Th2 immune responses [196].

Type 2 innate lymphoid cells (ILC2s) express MHCII, CD80, CD86, and OX40L and function as APCs for T cells. Interaction between ILC2s and T cells is facilitated by HSV-IL-2 and increases T-cell autoreactivity. Nevertheless, the lack of ILC2s lessens the impact of HSV-IL-2 on neurons, possibly by increasing chemokines. Neurons release chemokines to attract protective T cells. On the other hand, HSV-IL-2 can suppress the production of chemokines and thus limit T-cell activation. Such altered T cells may be involved in the demyelination of infected neurons [197].

Finally, different immune cells and markers play a beneficial role in the design of herpes vaccines, such as T cells, NK cells, NKT cells, and B cells. Other immune cells are deceived by viruses and shelter inside these immune cells, including neutrophils, monocytes, microglia, macrophages, DCs, MCs, and ILC2s. Both types of cells must be considered when evaluating future vaccines.

## 9. Conclusions

The development of an efficient herpes vaccine requires a deeper understanding of the relationship between immune response and the disease process. Current vaccines cannot overcome herpes hiding and latency in neurons. Adding to this are innate and adaptive immunity imbalance, abnormal levels of specific cell types, and the activation, exhaustion, and proliferation markers and cytokines. The aforementioned factors figure into the success of any vaccine. In this regard, the crosstalk between tissue-resident and humoral immune cells may prove a fruitful line of inquiry. Collectively, these factors have to be considered in immunological mathematical modeling to assess a successful vaccine, which has not been achieved yet. Ignoring one immune subset while assessing vaccination efficacy will result in failure and ineffective vaccines.
